# Postural Stability: The Role of Auditory Input in Normal Hearing Individuals and Older Adults with Hearing Loss

**DOI:** 10.22038/ijorl.2025.81193.3733

**Published:** 2025

**Authors:** Nikita Nanavati, Pragya Jain

**Affiliations:** 1 *School of Audiology and Speech Language Pathology, Bharati Vidyapeeth (Deemed to be University), Pune, Maharashtra, India.*

**Keywords:** Postural stability, Young adults, Auditory stimuli, Older adults with hearing loss

## Abstract

**Introduction:**

Balance integrates sensory and motor functions through the visual, somatosensory, and vestibular systems. Auditory inputs also contribute to spatial orientation, aiding postural control and stability. Exploring the effects of auditory stimuli on postural stability may reveal their therapeutic potential. So, current study is undertaken to study effect of auditory stimuli in maintaining postural stability in healthy young and older adults with age-related hearing loss.

**Materials and Methods:**

A total of 70 participants were divided into two groups: Group I consisted of 35 individuals aged 18-35 years with normal hearing, and Group II comprised 35 older adults aged 50-80 years with mild to moderate sensorineural hearing loss. Pure Tone Audiometry was performed, followed by a Modified Clinical Test of Sensory Interaction and Balance (mCTSIB) protocol and tandem gait on static postural stability, under four conditions (quiet, speech, natural environmental sounds, white noise), both with and without auditory stimuli.

**Results:**

Postural stability improved with natural sounds compared to quiet and other auditory stimuli in both young and older adults with age-related hearing loss. Healthy young adults consistently showed better postural stability than older adults in both quiet and auditory conditions.

**Conclusion:**

Auditory stimuli can effectively enhance postural stability in both young adults and older adults with age-related hearing loss, with more pronounced effect observed in older adults. Therefore, auditory stimuli can be effectively used to enhance postural stability, suggesting their potential utility in therapeutic interventions aimed at improving balance in individuals with age-related hearing impairments.

## Introduction

Balance is a process that integrates both sensory and motor functions. The primary sensory systems contributing to balance control are the visual, somatosensory, and vestibular systems (1). Postural stability is maintained through a dynamic integration of information from the vestibular, visual, and proprioceptive systems (2). 

The auditory system also influences postural control by modulating subsystems that affect stability in response to auditory stimuli. Various studies have examined how different auditory inputs such as quiet environments, natural sounds, speech stimuli, and white noise affect postural stability. 

Research has shown that auditory inputs significantly influence balance, with more complex auditory environments potentially enhancing stability. This improvement is achieved by providing directional cues through interaural time and level differences (3-5).

The aging process is linked to a decline in sensory system functionality, which contributes to an increased risk of falls among older adults. It has been observed that aging diminishes the efficiency of integrating vestibular, visual, and proprioceptive inputs, all of which are crucial for maintaining balance (6). Research has established that age-related declines in sensory and motor functions are common, and even minor hearing impairments are associated with a higher risk of falls (7). Presbycusis, or age-related hearing loss, impairs spatial awareness and reaction time, further elevating fall risks as hearing impairment becomes more prevalent with age (8). Studies have shown that older adults exhibit increased lateral body sway in response to the lateral movement of a sound source (9). Age-related changes in neuromuscular control and sensory input resolution contribute to greater sensitivity to noise and delays in processing sensory signals compared to younger individuals. Static posturography aids in understanding these mechanisms, providing insights into healthy balance, balance disorders, and the impacts of aging and auditory stimuli. 

Various studies have shown mixed results regarding the effects of auditory stimuli on postural stability, highlighting the need for further research to clarify the relationship between hearing and balance (10,11). While anecdotal evidence indicates that hearing loss may contribute to a sense of instability, there is still a lack of comprehensive research on the specific role of hearing in balance function.

Exploring interventions, it has been suggested that auditory feedback systems could assist individuals with balance issues due to hearing impairment, although some research indicates that exposure to non-specific auditory stimuli has minimal impact on postural stability (12,13). These findings emphasize the complex interplay between hearing and balance and underscore the necessity for additional research to deepen our understanding of this interaction.

Different auditory stimuli, such as white noise, natural environmental sounds, and narrative speech stimuli, may have diverse impacts on postural control. It is essential to explore the effects of these different auditory stimuli on postural stability to gain insights into their potential therapeutic applications. 

While auditory noise hasn't been previously employed to mitigate sway variability in older adults, it can present a promising avenue for compensating for diminished sensory feedback resulting from various deficits such as impaired vision, vestibular function, somatosensory perception, or auditory processing. This potential suggests that auditory interventions could address multiple contributing factors to postural instability in this demographic, offering a novel approach to enhancing balance control in older adults.To address these gaps in knowledge, this study aims to evaluate how auditory stimuli impact postural stability in both young adults with normal hearing sensitivity and older adults experiencing age-related hearing loss. Additionally, it seeks to investigate whether different auditory stimuli have varying effects and to compare their impacts on postural stability.

## Materials and Methods

This research is a cross-sectional prospective study. It received approval from the institutional ethics committee of Bharti Vidyapeeth (Deemed to be University) and was conducted in compliance with the university’s ethical guidelines. 

### Participants

A total of 70 participants, including both control and affected groups, were included in the study. Group I comprised 35 normal hearing individuals aged 18-35 years, serving as the control group. Group II consisted of 35 individuals aged 50-80 years with bilateral mild to moderate sensorineural hearing loss, designated as the case group. Participants included in the study had intact tympanic membranes, uncomfortable loudness levels up to 100 dBHL, and passed subjective vestibular screening tests, including the Romberg test and past pointing test. 

Exclusion criteria for both groups encompassed individuals with a history or current presence of middle ear issues, neurological conditions like epilepsy or peripheral neuropathy, uncontrolled systemic diseases, retro-cochlear pathology, recent injuries, or musculoskeletal disorders. Additionally, participants who participated in activities or training known to affect balance, such as yoga or classical dance, were excluded from the study.

### Instrumentation and Tool

White noise was generated through the Audacity software and a narrative speech sample is recorded through Audacity software to assess postural stability for different stimuli. The natural environmental sound was taken from the Pixabey.

The posturography instrument used for the study is EquiPoise Computerised Stabilometric Posturography by Equidor Medtech LLP. Postural sway measurements during quiet standing were recorded using a force platform measuring 60 cm by 60 cm by 11 cm. The force plate registers vectors across three dimensions: anterior-posterior (AP), medial-lateral (ML), and vertical components (Fx, Fy, Fz). Postural stability outcome variables are obtained from measurements of both anteroposterior and lateral positions. 

### Procedure: 

All participants received comprehensive information about the study, covering its objectives, procedures, potential risks, and benefits. Prior to their inclusion, each participant gave written informed consent, confirming their full understanding and willingness to participate. All subjective tests were conducted in a well-lit room with ambient noise levels maintained within the permissible limits (ANSI S3.1, 1999).

 A comprehensive case history was obtained from all participants, including relevant details about their medical history, hearing and vestibular symptoms, and any other pertinent information for the study. Pure Tone Audiometry, encompassing octave frequency ranges from 0.25kHz - 8 kHz for air conduction and 0.25 Hz - 4 kHz frequency for bone conduction thresholds, was performed using Carhart and Jerger (1959) modification of Hughson & Westlake (1985) ascending method. Vestibular screening including the Romberg test and past pointing was done to assess balance and the function of the vestibular system.

A Modified Clinical Test of Sensory Interaction and Balance (mCTSIB) protocol on static postural stability (escalating sensory denial) was assessed under the following conditions, with each condition recorded for 30 seconds: stable surface with eyes open, stable surface with eyes closed, foam surface with eyes open, foam surface with eyes closed. Also, tandem stance with eyes open, and eyes closed (escalating motor complexity) was also performed on static posturography. These various conditions are structured to evaluate an individual's ability to sustain stability under different sensory inputs, including vision, proprioceptive, and vestibular systems, and physical circumstances. By systematically altering these conditions, the study can identify specific factors that influence balance and postural control, providing a more detailed understanding of how individuals adapt to environmental changes to maintain stability.

All these tasks were performed under four conditions using static posturography: quiet environment, white noise, speech and natural environmental sound. The stimuli were presented in random order. During tests involving auditory stimuli, participants were instructed to concentrate on the sound being presented. All stimuli were delivered through a calibrated loudspeaker at 65 dB SPL from a 0-degree azimuth at a distance of 3 feet. The parameters measured using a stabilometer included sway velocity, maximum sway distance, sensory functional score, and the ratio of normal to tandem stance.

### Statistical analysis:

The collected data was organized in an Excel spreadsheet and analyzed using suitable statistical methods based on the data distribution to meet the objectives of the current study. Statistical analysis was carried out using IBM SPSS version 29. All results were presented in tabular formats. The normality of the data was assessed using the Shapiro-Wilk test, which indicated a non-normal distribution (p<0.05). Consequently, non-parametric tests were employed for the analysis. Descriptive statistics, including the mean, median, standard deviation, and interquartile ranges, were calculated. The Wilcoxon signed-rank test was used for within-group comparisons of postural stability in quiet environments versus those with natural environmental sounds, speech sounds, and white noise in both groups. Friedman's test was applied to compare responses to different auditory stimuli across the groups. The Mann-Whitney U test was utilized to assess the impact of auditory stimuli on postural stability between healthy young adults and older adults with age-related hearing loss.

## Results

Descriptive statistics were computed for participant demographics including age, gender distribution, and pure tone averages. The mean age for participants with normal hearing was 25 years ± 5.24. The mean age for participants with mild to moderate sensorineural hearing loss was 66 years ± 7.72. The distribution of males and females across these groups was as follows: normal hearing (10 males, 14 females), mild to moderate sensorineural hearing loss (16 males, 8 females). The average pure tone audiometry results were 11.29 dB (right ear) and 11.19 dB (left ear) for normal hearing, 37.91 dB (right ear) and 40.09 dB (left ear) for mild to moderate sensorineural hearing loss. Furthermore, median and interquartile ranges were computed for all the auditory stimuli for healthy young adults and older adults with age related hearing loss. In the present study, postural stability was assessed with and without auditory stimuli in young adults and older adults with age-related hearing loss. In young adults, significant differences were observed in postural stability when comparing non-auditory versus different auditory stimuli, as shown in Table 1. For natural sounds and speech stimuli, significant differences (p<0.05) were noted on a stable surface with eyes closed. For white noise, significant differences (p<0.05) were found on a cushioned surface with eyes closed and during tandem gait with eyes open.

**Table 1:** Wilcoxon Signed Rank Test results to compare postural stability in the quiet situation with auditory stimuli in healthy young adults.

**Conditions**	**Parameters**	**Quiet-Natural**	**Quiet-Speech**	**Quiet-White**
Stable surface eyes closed	Sway velocity	0.27	0.84	0.17
	Maximum sway distance	0.009	0.26	0.09
Cushioned surface eyes open	Sway velocity	0.84	0.96	0.47
	Maximum sway distance	0.84	0.59	0.70
Cushioned surface eyes closed	Sway velocity	0.85	0.046	<0.001
	Maximum sway distance	0.12	0.40	0.97
Tandem gait eyes open	Sway velocity	0.30	0.07	0.57
	Maximum sway distance	0.29	0.18	0.012
Tandem gait eyes closed	Sway velocity	0.96	0.30	0.25
	Maximum sway distance	0.74	0.42	0.49
Sensory Functional score	Vestibular system	0.85	0.39	0.90
	Somatosensory	0.64	0.18	0.79
	Normal to tandem stance ratio	0.44	0.41	0.41


Similarly, in older adults with age-related hearing loss, significant differences in centre of pressure were observed when comparing a quiet environment to different auditory stimuli, as shown in Table 2. For natural sounds, significant differences (p<0.05) were noted on a cushioned surface with both eyes open and closed. For speech stimuli, significant differences (p<0.05) were observed on a cushioned surface with eyes open and closed, as well as on a stable surface with eyes closed. For white noise, significant differences (p<0.05) were found during tandem gait with eyes closed.

**Table 2:** Wilcoxon Signed Rank Test results to compare postural stability in the quiet situation with auditory stimuli in the older adults with age-related hearing loss.

**Conditions **	**Parameters**	**Quiet-Natural**	**Quiet-Speech**	**Quiet-White**
Stable surface eyes closed	Sway velocity	0.97	0.01	0.96
	Maximum sway distance	0.92	0.18	0.27
Cushioned surface eyes open	Sway velocity	0.83	0.34	0.76
	Maximum sway distance	0.01	0.98	0.09
Cushioned surface eyes closed	Sway velocity	0.21	0.002	0.21
	Maximum sway distance	0.004	0.006	0.12
Tandem gait eyes open	Sway velocity	0.93	0.90	0.76
	Maximum sway distance	0.99	0.66	0.71
Tandem gait eyes closed	Sway velocity	0.81	0.89	0.04
	Maximum sway distance	0.89	0.96	0.12
Sensory Functional score	Vestibular system	0.36	0.37	0.85
	Somatosensory	0.53	0.46	0.25
	Normal to tandem stance ratio	0.98	0.47	0.96

n=24; significant difference (p<0.05)

Postural stability was compared across various auditory stimuli in both the groups. For healthy young adults, when center of pressure was compared across different auditory stimuli, the analysis demonstrated a significant difference (p<0.05) between natural stimuli and speech stimuli across various conditions, such as cushioned surface with eyes open and closed, as well as tandem gait with eyes open and closed. Furthermore, a significant difference (p<0.05) was noted between natural stimuli and white stimuli, particularly in cushioned surface conditions with eyes closed. Whereas for older adults with age-related hearing loss, significant differences (p<0.05) were observed between natural stimuli and speech stimuli in cushioned surface with eyes open conditions. Also, significant difference (p<0.05) were found between natural stimuli and white stimuli in cushioned surface with eyes closed, tandem gait with eyes closed, and between speech stimuli and white stimuli in cushioned surface with 

eyes open and closed conditions. Additionally, postural control was compared across different auditory stimuli between young adults and older adults with age-related hearing loss. Statistically significant differences (p<0.05) were observed for a quiet environment as well as in different auditory stimuli as shown in Table No 3. In quiet environment as well as for natural stimuli, significant differences (p<0.05) were observed in the stable surface eyes closed, cushioned surface eyes open and closed, and normal to tandem stance ratio conditions between the two groups. For white noise stimuli, statistically significant differences (p<0.05) were observed in the stable surface eyes closed, cushioned surface eyes closed, and normal to tandem stance ratio conditions. Furthermore, for speech stimuli, statistically significant differences (p<0.05) were noted in the cushioned surface eyes open and normal to tandem stance ratio conditions between the two groups.

**Table 3:** Mann Whitney U test results to compare the quiet environment, natural environment, speech sound and white noise stimuli using postural stability between young adults and older adults with age related hearing loss.

		**Quiet** **environment**		**Natural environment**		**White** **Noise**		**Speech** **sound**	
**Conditions**	**Parameters**	**U**	**Sig.**	**U**	**Sig.**	**U**	**Sig.**	**U**	**Sig.**
Stable surface eyes closed	Sway velocity	149.50	0.003	114.5	0.00	95.50	0.00	238.50	0.31
	Maximum sway distance	280.00	0.86	193.5	0.05	209.00	0.09	211.00	0.11
Cushioned surface eyes open	Sway velocity	256.50	0.51	220.00	0.16	281.00	0.88	240.50	0.33
	Maximum sway distance	153.50	0.004	197.00	0.05	226.00	0.20	183.00	0.02
Cushioned surface eyes closed	Sway velocity	256.00	0.50	276.00	0.80	266.00	0.65	286.00	0.97
	Maximum sway distance	133.50	0.001	153.50	0.004	180.00	0.02	194.00	0.04
Tandem gait eyes open	Sway velocity	261.50	0.59	280.00	0.87	271.00	0.73	218.00	0.14
	Maximum sway distance	238.00	0.30	253.00	0.47	269.00	0.70	276.00	0.81
Tandem gait eyes closed	Sway velocity	275.50	0.80	283.00	0.92	231.00	0.23	285.00	0.95
	Maximum sway distance	274.00	0.77	256.00	0.51	287.00	0.99	241.00	0.33
Sensory Functional score	Vestibular system	228.00	0.21	238.50	0.31	263.00	0.61	232.50	0.25
	Somatosensory	281.00	0.88	229.50	0.22	254.00	0.47	285.50	0.96
	Normal to tandem stance ratio	115.00	0.00	84.00	0.00	94.00	0.00	141.00	0.002

Group I: n= 24; Group II: n=24; W= Mann-Whitney U test values; *= significant difference (p<0.05)

## Discussion

In present study, postural stability was enhanced in both young and older adults with hearing loss when exposed to various auditory stimuli, including natural environmental sounds, speech, and white noise. This finding aligns with previous research on healthy young adults, which demonstrated that natural environmental sounds can improve postural stability, particularly under conditions of impaired vision (14,15). The literature also indicates that continuous sound provides greater postural benefits than interrupted noise (16). This supports other research suggesting that white noise can reduce postural fluctuations (17). Additionally, it has been reported that participants who wore hearing protection, blocking stationary pink noise exhibited greater postural sway (19). Moreover, richer auditory stimuli, such as speech, may be even more beneficial than static sounds or silence (18,5). Environmental sounds provide spatial cues through consistent auditory signals, aiding in spatial orientation and stability by reducing the cognitive load required to process spatial information, which can be especially useful in environments with limited visual or proprioceptive input (5). Speech can have a mixed effect on balance: familiar speech can serve as an auditory cue aiding spatial orientation, whereas complex speech, particularly for older adults or those with cognitive deficits, can be distracting and disrupt balance (26). White noise can be helpful for masking disruptive sounds and improving focus on balance, although it may not offer the same richness of spatial cues as natural sounds (11).

In the current study, postural stability across various auditory stimuli was assessed in both young adults and older adults with hearing loss. The findings indicate a significant difference between responses to natural sounds compared to speech and white noise, with natural environmental sounds leading to enhanced postural control. This aligns with previous research which indicated that natural sounds significantly improve postural control compared to other auditory stimuli, such as white noise or speech, especially in conditions where visual input is limited or absent (20). These findings suggest that natural sounds are beneficial for maintaining postural control and reducing sway, making them an effective tool for improving balance in various populations. Also in literature (25), the multifaceted relationship between auditory cues and postural stability has been reported. Auditory information plays a role in constructing a mental representation of the surrounding environment, thereby influencing balance control. Importantly, the type of auditory stimulus significantly impacts postural stability, likely due to its interaction with sensory and cognitive systems. For instance, speech stimuli require more complex cognitive processing while potentially offering spatial cues. However, this increased cognitive demand can also negatively impact balance (20). In contrast, natural sounds necessitate less cognitive effort and provide valuable environmental context, potentially improving balance due to their calming properties and contribution to well-being. Finally, the emotional content of speech can influence balance, promoting stability or instability depending on the emotional tone (20).

Current study investigated the impact of auditory cues on postural stability in both younger and older adults with age-related hearing loss across various environmental conditions. In quiet, younger adults demonstrated superior postural control compared to older adults, particularly during challenging tasks such as stable surface eyes closed, cushioned surface eyes open and closed, and normal to tandem stance ratio conditions. These findings align with previous research, which reported increased postural sway in older adults during complex balance tasks (21). They observed age-related differences highlight the significant role of sensory input changes in postural control. Consistent with prior research (22), older adults with hearing loss exhibited greater difficulty maintaining balance compared to younger adults, especially in the absence of visual cues. This highlights significant age-related differences in postural stability, particularly under conditions where sensory input is compromised. This emphasizes the compounding challenges posed by sensory impairments in the elderly population. Additionally, studies have shown that older adults tend to exhibit higher temporal correlations in postural sway patterns compared to younger adults, influencing phenomena like stochastic resonance when using vibrating insole (23,24).

Also, significant differences were observed between young adults and older adults with age-related hearing loss in response to different auditory stimuli. Findings in the present study suggest that natural environmental sound, can be beneficial for improving postural stability in older adults with hearing loss. It was observed that exposure to natural sound led to a greater reduction in sway variability for older adults compared to young adults across a variety of conditions, including cushioned surface eyes open and closed, and normal to tandem stance ratio condition. These results indicate that auditory cues may have a more significant stabilizing effect on the postural control of older individuals (20). These results underscore the heightened vulnerability of older individuals to postural instability, particularly in the absence of optimal sensory input. Furthermore, our study aligns with previous findings (5) highlighting the potential of auditory cues to mitigate postural sway in older adults. The observed reduction in sway variability with the introduction of auditory stimuli suggests a promising avenue for developing targeted interventions to enhance balance and prevent falls in this population. However, further investigation is warranted to explore the optimal auditory environments and stimulus characteristics for maximizing postural benefits.

Also, in present study, young adults showed better postural stability on cushioned surfaces with eyes open, in both normal and tandem stance conditions, compared to older adults with age-related hearing loss when exposed to speech stimuli. Similarly, it has been reported that speech stimuli can help to reduce lateral sway and improve balance by narrowing the lateral boundaries of postural stability (26). However, another study found that speech stimuli, whether meaningful or not, improved postural stability more in older adults (27). Similar results were observed for white noise in the present study, where young adults demonstrated better postural stability compared to older adults with age-related hearing loss. Older adults with hearing loss exhibited a decrease in sway variability compared to young adults. This suggests that the use of noise may have a stabilizing effect on the postural control of older adults. These findings are consistent with those reported in the literature (28). Older adults with age-related hearing loss experience greater declines in postural stability compared to healthy young adults, likely due to the deterioration of bodily functions and the vestibular system. Aging affects the sensory hair cells in the cochlea and auditory nerve fibers, reducing the ability to process sounds, especially high-frequency ones, impacting spatial hearing and auditory mapping essential for posture. Additionally, aging diminishes the brain's capacity to integrate auditory information with other sensory inputs, impairing balance. Declines in the vestibular system and reflexes, as well as difficulties in integrating vestibular signals with other sensory inputs, further challenge balance in older adults. In quiet settings, both young and older adults rely on visual and proprioceptive cues, but young adults integrate these cues more efficiently. In natural environments, young adults benefit from spatial auditory cues, while older adults are limited by reduced auditory processing. Speech stimuli provide directional cues for young adults but are challenging for older adults with hearing loss. White noise is generally neutral for young adults, providing no significant spatial cues. However, older adults might find it disorienting and harder to filter out, potentially increasing instability. 

Overall, age-related decline in auditory and vestibular systems reduce the effectiveness of auditory cues in aiding balance. Similarly, extensive research has established a strong link between hearing loss in older adults and an increased risk of imbalance and falls. The significance of our study lies in demonstrating how auditory cues, particularly natural environmental sounds, can enhance postural stability. Our findings highlight the potential of auditory-based interventions tailored to older adults with hearing loss by fitting hearing aids, potentially reducing fall incidence and improving overall quality of life. Hearing aids can amplify environmental sounds, improve spatial awareness, and enhance the brain’s ability to process auditory cues, thereby supporting balance and stability. The integration of hearing aid technology with balance training programs could offer a comprehensive approach to mitigating fall risks in this population.

## Conclusion

The test findings suggest a significant difference in postural stability with and without auditory stimuli for both young adults and older adults with age-related hearing loss. It can be concluded that postural stability was better with natural stimuli compared to a quiet environment, white noise, and speech stimuli. Also young adults exhibited significantly better postural stability as compared to older adults. These results suggest that auditory stimuli may be effectively utilized to enhance postural stability in individuals with age-related hearing loss. This study enhances our understanding of the role of auditory stimuli in maintaining balance and explores the relationship between meaningful and non-meaningful auditory stimuli in postural stability. Future research can investigate the effects of varying degrees of hearing loss on postural stability in older adults and the impact of auditory cues on maintaining postural stability across different degrees of hearing loss in this population.
